# The Association between Four Citation Metrics and Peer Rankings of Research Influence of Australian Researchers in Six Fields of Public Health

**DOI:** 10.1371/journal.pone.0018521

**Published:** 2011-04-06

**Authors:** Gemma Elizabeth Derrick, Abby Haynes, Simon Chapman, Wayne D. Hall

**Affiliations:** 1 Sydney School of Public Health, Sydney Medical School, The University of Sydney, Sydney, New South Wales, Australia; 2 Instituto de Politcas y Bienes Publicos, Centro de Ciencias Humanas y Sociales, Consejo Superior Investigaciones Cientificas, Madrid, Spain; 3 Centre for Clinical Research, The University of Queensland, Brisbane, Queensland, Australia; Institute of Marine Research, Norway

## Abstract

Doubt about the relevance, appropriateness and transparency of peer review has promoted the use of citation metrics as a viable adjunct or alternative in the assessment of research impact. It is also commonly acknowledged that research metrics will not replace peer review unless they are shown to correspond with the assessment of peers. This paper evaluates the relationship between researchers' influence as evaluated by their peers and various citation metrics representing different aspects of research output in 6 fields of public health in Australia. For four fields, the results showed a modest positive correlation between different research metrics and peer assessments of research influence. However, for two fields, tobacco and injury, negative or no correlations were found. This suggests a peer understanding of research influence within these fields differed from visibility in the mainstream, peer-reviewed scientific literature. This research therefore recommends the use of both peer review and metrics in a combined approach in assessing research influence. Future research evaluation frameworks intent on incorporating metrics should first analyse each field closely to determine what measures of research influence are valued highly by members of that research community. This will aid the development of comprehensive and relevant frameworks with which to fairly and transparently distribute research funds or approve promotion applications.

## Introduction

There are two broad approaches in evaluating research and researchers: traditional methods of peer assessment used for publishing, grant proposals and promotion purposes; and the newer use of citation metrics for comparative evaluation. Given that metrics are easily used and transparent, their use as an adjunct or potential replacement for the more lengthy, costly, subjective and often erratic process of peer review is under active consideration [1]. Despite the imperfect nature of peer review, it is accepted as the best method we have for making decisions about the worth or potential of a candidate, project or paper. Thus, if metrics are to be a useful evaluation method they must be seen to deliver similar results as the considered judgement of peers [2]. This research, therefore, sought to investigate how peer assessment corresponds with four different citation metrics.

In grant, promotional and institutional ranking exercises, assessments are typically undertaken by a small number of assigned peers or by relatively small peer panels. The process is often shrouded in secrecy and judgments are based on a series of intellectual and social processes that may be mediated by factors other than the quality, importance or impact of the research under evaluation [3]. For example, evaluators may be influenced by political and social pressures within the scientific community, such as possible repercussions of their decisions affecting their work and that of colleagues. In addition, peer reviewers tend to evaluate work in terms of their own research interests and may not possess the granular knowledge required for expert analysis outside their immediate field nor the broader knowledge needed for making ‘big picture’ judgements about the importance of research. Finally, the reliability of peer review is questionable [4]. As Langfeldt (2001) argues, ‘while there is a certain set of criteria that reviewers pay attention to – more or less explicitly – these criteria are interpreted or operationalized differently by various reviewers' [5].

Citation metrics offer potential cost and efficiency advantages over the sometimes arduous and often expensive process of peer review. When used and calculated properly, metrics can provide objective, transparent, replicable and comparable information that is based on the aggregated behaviour of large numbers of (citing) researchers rather than on the views of a small group of peers [6]. Metrics can be simple (e.g. citation counts or citations per paper) or more complex such as the *h*-index and its variants. These metrics may be used to complement peer review or to validate peer review outcomes or, more controversially, to replace peer review. Moed (2007) argues that the challenge for the future of research evaluation lies in the intelligent combination of advanced bibliometrics with transparent peer review processes. Using the two methods collaboratively would allow for one method to compensate for the limitations of the other [7]. The combination of peer review and metrics also reflects the broader trend of evaluation frameworks towards the use of mixed methods, both qualitative and quantitative, when evaluating research and researchers.

Traditionally, peer review has been used as the ‘gold standard’ in validating bibliometric indicators. Whenever a new bibliometric indicator is proposed there is always the question of its convergent validity: how is it related to other (advanced) bibliometrics indicators and to assessments by peers [2]? The assumption is that a new bibliometric indicator only has the potential to be a useful measure for evaluating researchers if it also correlates strongly with peer assessments.

Studies assessing the level of agreement between research metrics and peer review outcomes have produced mixed results. Bornmann et al. (2008) investigated the relationship between different metrics and a researcher's success in grant or fellowship applications decided by peer review [2], [8]. Although the exact assessment criteria used for the allocation of grants and fellowships were unclear, there were high correlations between a number of metrics and the outcome of the fellowship applications. Other studies have shown that the average h-index values of accepted and rejected applicants for biomedicine researcher fellowships differed significantly [8], [9], [10]. Van Raan (2006) found that the h-index was positively correlated with peer judgements for 147 Dutch chemistry research groups [11].

It is unclear whether the metrics used in these studies (*h*-index and its variants) can be appropriately applied to all fields, research types and researchers. Bornmann (2008a; 2008b; 2008c; 2009) analysed the biomedical field, and other studies have found similar correlations in fields such as physics [2], [8], [9], [10], [12], but no studies have compared the relationships between metrics and peer evaluations in more applied fields. This may be because inter-field comparisons are difficult to conduct and the relationship between metrics and outcomes of peer review may be more indicative of the nature of the field, rather than the accuracy of the metrics per se. It may be that fields differ in their relationship between these metrics and peers' views of research excellence and influence.

The *h*-index is a popular metric that is increasingly used by researchers and funders. Loosely defined, a researcher with an *h*-index of 10 has at least 10 publications each with 10 or more citations. A researcher with an *h* of 20 has a minimum of twenty papers with twenty citations, and so on. The *h*-index for any author can be calculated automatically using either, or a combination of, the Web of Science (WoS), Scopus, and Google Scholar. The accuracy of the calculation will depend on what publications a database covers and the type of analyses performed [13]. Because it is increasingly being used to evaluate grant proposals and promotion applications, researchers seek to maximise their *h*-index by using a combination of databases. It is therefore essential that any study calculating and comparing researchers' *h*-indices uses the same publication database platform to ensure comparability [7].

The strength of the *h*-index is that it provides a single number that describes a researcher's publication performance by combining the number of publications with a measure of their citation frequencies. However, the *h*-index may not fairly compare researchers from different fields of research, because of large variations in publication type and frequency and citation volumes between fields. Comparisons of researchers at different stages of their careers are also problematic because later career researchers will generally have larger *h*-indices than early career researchers. Moreover, as the *h*-index can only increase over time, it is insensitive to changes in a researcher's performance and is only weakly correlated with the total number of citations their body of work has received [14].

Another, more pertinent, criticism of the *h*-index is that it does not adequately summarise citation patterns. Researchers who have published a few highly cited articles and many rarely cited articles will have an *h*-index that is right or left skewed and which may misleadingly represent their publication output. As the *h*-index provides an incomplete picture, it should ideally be used to evaluate researchers in coordination with other research metrics, which describe different aspects of research output (Bornmann 2008a). A number of variants have been proposed to address the limitations of the *h*-index and provide alternative views of a researcher's research performance. Bornmann (2008a; 2009) suggests that a complete evaluation of researchers' publication outputs can be achieved by combining two indices; one that describes the most productive core of a researcher (such as the *h*-index) and one that describes the impact of the papers in the core (such as the *m*-index) [9], [10].

The aims of this study were: (1) to identify relevant metrics that could be used in conjunction with peer review to assess researchers; and (2) to evaluate the relationship between peer assessment and selected citation metrics among Australian-based public health researchers in six fields. Public health is an interesting field in which to assess these relationships because it is an applied field in which research influence may not be well captured by citation indices for peer reviewed publications. Accordingly, this paper evaluated the relationships between (1) the *h*-index and its main variants and (2) rankings of researchers' influence as assessed by research peers in the same field. It also provided a comparison of the correlations between peer review outcomes and bibliometric indicators in each of six public health fields.

## Methods

### Participant recruitment

Australia-based participants from six different fields of public health research (Alcohol, Drugs, Tobacco, Skin Cancer, Injury and Obesity) were asked to nominate up to five Australia-based researchers whom they considered to be *‘most influential in shaping any aspect of policy or programs, legislation, clinical practice, or public understanding’* within their respective fields. ‘Influence’ was thus not restricted to scientific impact within the research community. This construct attempted to harness some of the more elusive social considerations that would be likely to inform peer assessment of grant or promotional candidates. A full description of participant recruitment and study methods is available elsewhere [15], [16].

We tested the hypothesis that there would be a strong association between the total number of nominations an individual received from their peers (‘influential researcher votes’ or IRVs) and these citation metrics. Only researchers who had received at least one IRV were included in the analysis.

### Ethics

The study engaged a large proportion of active Australian public health researchers and provided a ranked distribution of peer esteem in each of the six fields.

An email explaining the project and requesting a copy of each participant's publication list was sent to all researchers who received at least one vote from their peers as an influential researcher (n = 182). Many of the researchers approached were unaware of their *h*-index. Some were concerned that it might be used to their disadvantage. In the majority of cases, participants were aware of the *h*-index but did not know how to calculate it. Participation in this project was thus encouraged by the offer of having one's *h*-index calculated. In addition, the research team reassured each participant that this information would be treated confidentially and not used in publications to characterise their individual research output. Informed written consent to calculate and use their publication metrics was obtained from each of the participants via email.

This study was cleared by the Behavioural & Social Sciences Ethical Review Committee (BSSERC) at The University of Queensland and the Human Research Ethics Committee (HREC) of The University of Sydney in accordance with the National Health and Medical Research Council's guidelines, protocol number 2009000340.

### Indices investigated

The *h*-index was supplemented with a series of related indicators. These included a combination of metrics that evaluated different aspects of research impact taking into account the number of publications, citations and career length. A full list of the metrics used in this study and their definitions are described below.

#### The h-index

Hirsch's index depends on both the number of a scientist's publications and the number of citations for each paper. It is defined as *‘a scientist has index h if h of his or her Np papers have at least h citations each and the other (Np - h) papers have ≤h citations each’*.[17]. The ‘Hirsch core’ contains the first h-papers of a researcher's publication output [18]. The concept of the Hirsch core is important for the description of the indices described below.

#### The m-index

The *m*-index is defined as the median number of citations received by papers that have a ranking that is equal to or smaller than *h* [9]. The median is used instead of the average because the distribution of citation counts is usually highly skewed. The *m*-index has been found to discriminate better between approved and rejected post doctoral fellowship applicants than the *h*-index [9].

#### The m-quotient

To take account of the fact that a researcher's *h*-index is approximately proportional to their career length [19], we calculated the *m*-quotient by dividing the *h*-index by the number of years since the author's first publication (*n*). This quotient was particularly pertinent in the current study as both senior and more junior researchers were included.

#### The q^2^-index

The *q2* index is the geometric mean of the *h*-index and the *m*-index, defined as the square root of the product of the *h-* and *m-* indices [20]. This index captures both the number of papers (quantitative dimension) and the impact of the papers (qualitative dimension) in a researcher's productive (*h*) core [20].

### Index calculation

Once the publications lists were received from participants, indices were calculated on researchers' entire body of published work rather than solely on publications in the field within which they were nominated as influential.

Using WoS, the number of citations for each paper was recorded. A further, cited reference search was conducted for those publications listed (such as books and other grey literature) that were not found by the general WoS search. If citations for a publication were not found using either of these methods, it was assumed that the publication had not achieved enough visibility within the research literature to affect the researcher's *h*-index. A researcher's *h*-index was manually calculated using Hirsch's formula from the list of the total publications and their citations [17].

If no reply to the email enquiry was received from a researcher, a preliminary blanket search for publications was performed using WoS. A preliminary *h*-index was then calculated and this calculation, together with the publication list used in its calculation, was sent to the researcher for confirmation or correction. Missing publications noted by the researcher were then included in a subsequent calculation. In all cases, an *h*-index calculation was only included in the analysis once the relevant researcher confirmed his or her publication list. In total, 176/182 (96.7%) researchers provided the research team with their CV and publication lists to calculate their respective indices. The remaining 3.3% of researchers' indices were calculated from their C.V independently, with the list of publications and citations sent to the researcher for approval and to alert the team of any missed publications that may change the index calculation. This process ensured that all eligible researchers participated in the project.

### Statistical analysis

SPSS and SAS were both used to analyse the data. Spearman's rho (rank correlation) was used for the correlations and significance was assumed when p<0.05.

## Results

### Characteristics of nominated influential researchers

A total of 182 public health researchers received at least one IRV in six fields of interest: Alcohol (31); Drugs (37), Injury (30); Skin cancer (29); Tobacco (19) and Obesity (36). Overall, the majority were men (68.1%), 64.8% were at professorial level and the majority were employed by universities (63.2%) or independent research organisations (17%).

### Peer-rankings of researcher influence

**Figure 1:**
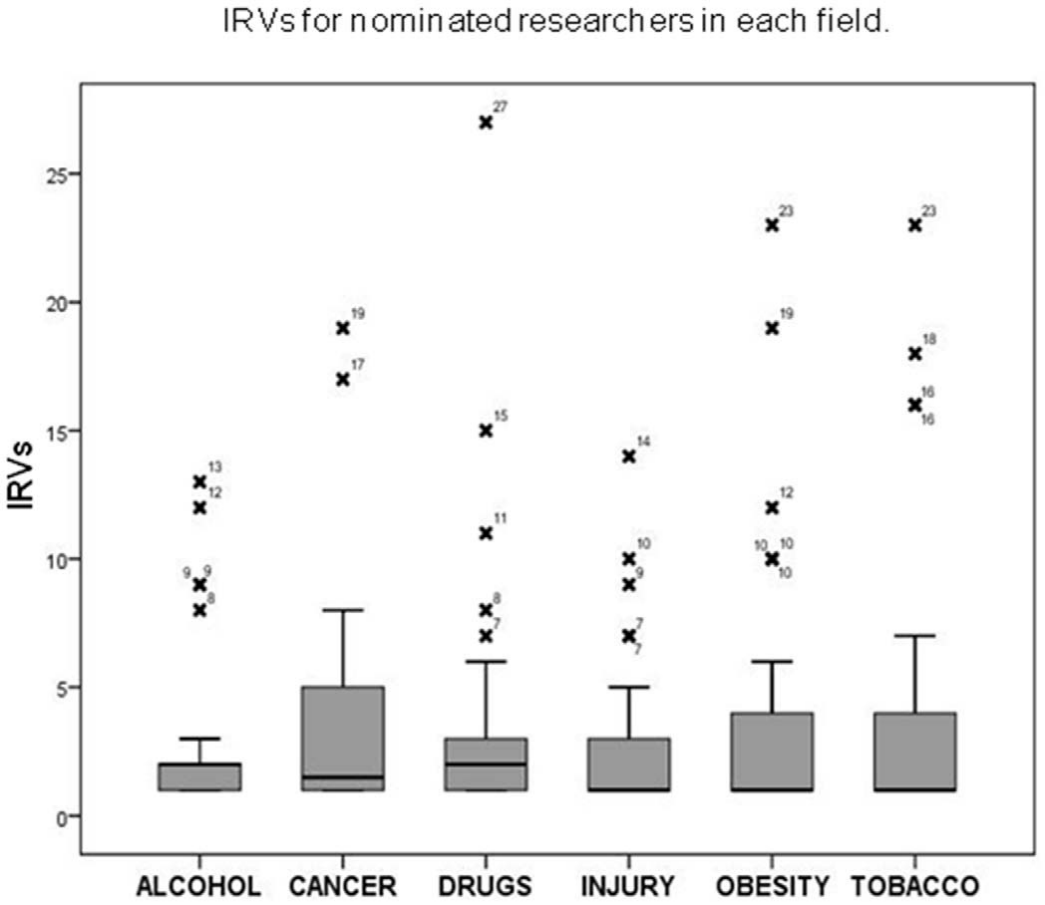
IRVs for nominated researchers in each field.

The box plot in Figure 1 shows the distribution of rankings made by participating researchers in each of the six fields. Each cross represents a nominated researcher with their corresponding number of received votes also shown. The line in the middle of each box represents the median of the sample. In all six fields, a large number of the 182 researchers (n = 94, 51.6%) received only one vote. In the tobacco field the top six researchers attracted 75% of all votes (σ^2^ = 39.9). The same trend was seen in Skin Cancer (σ^2^ = 20.0), Drugs (σ^2^ = 22.1) and Obesity (σ^2^ = 24.8) fields. Only in the Injury field (σ^2^ = 9.7) did less than 50% of researchers vote for the person who was ranked first.

### Index characteristics by field

Averages for each of the indices were calculated and compared across the six public health areas using ANOVA (see Table 1). There were no significant differences between fields for the four indices of interest but they differed in the average number of years since researchers' first publication (p = 0.03, p<0.05).

**Table 1:** Comparison of index means over 6 public health fields.

	*m*-INDEX	*m*-QUOTIENT	*h*-INDEX	*q^2^*-INDEX	YEARS
ALCOHOL	33.7	0.73	19.2	25.3	28.4
CANCER	49.6	0.90	26.8	36.3	29.8
DRUGS	35.8	0.85	19.8	26.4	24.3
INJURY	40.7	0.87	21.3	29.2	25.7
OBESITY	40.0	1.02	20.9	28.7	22.7
TOBACCO	40.9	0.80	23.1	30.7	29.3
P-value	0.15	0.11	0.23	0.17	0.03

### Correlation with peer rankings of research influence

Table 2 shows Spearman rank correlation coefficients (r) for the correlations between IRVs and the *h*-index, *m*-index, *m*-quotient, *q^2^* index and the number of research active years. The number of IRVs, irrespective of field, was found to significantly correlate with all bibliometric indicators of interest in this study. As the correlation coefficients were small, 95% confidence intervals were constructed around the means to indicate the degree of uncertainty around the estimated correlations within fields.

**Table 2:** Correlation of Evaluation metrics with Votes of Researcher Influence (IRVs).

		*m*-INDEX	*m*-QUOTIENT	*h*-INDEX	*q^2^*-INDEX	YEARS
**OVERALL**	***r***	0.21*	0.22*	0.26*	0.25*	0.04
	***P-value***	<0.01	<0.01	<0.01	<0.01	0.53
**ALCOHOL**	***r***	0.40*	0.25	0.40*	0.42*	0.13
	***P-value***	0.02	0.17	0.03	0.02	0.46
**SKIN CANCER**	***r***	0.56*	0.48*	0.59*	0.60*	0.36
	***P-value***	<0.01	<0.01	<0.01	<0.01	0.06
**DRUGS**	***r***	0.18	0.36*	0.32*	0.25	0.03
	***P-value***	0.28	0.03	0.05	0.13	0.83
**INJURY**	***r***	−0.33	−0.02	−0.32	−0.34	−0.34
	***P-value***	0.07	0.91	0.09	0.05	0.06
**OBESITY**	***r***	0.42*	0.27	0.45*	0.49*	0.14
	***P-value***	0.01	0.11	<0.01	<0.01	0.40
**TOBACCO**	***r***	0.00	−0.03	0.05	0.03	0.04
	***P-value***	0.99	0.90	0.82	0.88	0.86

*Significant correlation at the p<0.05 level.

There were some interesting differences between fields. For the tobacco field there were no significant correlations between the number of IRVs and any of the bibliometric indicators. By contrast, in the skin cancer field there were significant positive correlations with all bibliometric indicators. The other fields showed significant correlations with different bibliometric indicators.

Closer examination of the negative correlation in the injury field showed a high proportion of researchers with a high *h*-index only receiving one IRV. Likewise, many of the researchers in this field who received a large number of IRVs recorded -indices that were relatively low compared to the entire Injury field. When we removed the researcher with the most IRVs, the correlation coefficient was still negative but no longer statistically significantly different from zero (r^2^ = 0.07; p = 0.675).

Finally, despite a significant difference found between fields for the mean number of research active years (Table 1), there were no significant correlations between this and the number of IRVs. Correlations were found between the *m*-quotient (which takes into account the number of research active years) and the number of IRVs for skin cancer (r = 0.48, p<0.01; CI_.95_ = 0.14–0.73) and drugs (r = 0.36, p = 0.02; CI_.95_ = 0.05–0.62).

### Determining the predictive power of individual and mixed metrics

We used multiple linear regression to determine whether the bibliometric indices, when used in combination, significantly predicted a researcher's rating by peers of their influence. The aim was to identify particular combinations of metrics that, when used together, would provide a more complete picture of a researcher's influence as determined by the number of peer votes.

Specifically, we used a combination of metrics depicting the quality and quantity of the *h*-core as recommended by Bornmann et al. (2008a). This method was used because many of the metric calculations were based on the *h*-index; therefore combinations of two metrics from the same basis were not expected to contribute to a combination's significance. For the purposes of this analysis, two distinct metrics were chosen—the *h*-index and the *m*-index—in order to investigate the potential value of adding the m-index when considering a researcher's overall influence as measured by his peers. Simple regression was preferred as a high correlation was found between the *h*- and the *m*-index (r = 0.91, p<0.001). This analysis was conducted in response to Bornmann's (2008a) recommendation outlined above.

Irrespective of field, the regression model that included the *h*-index and *m*-index was found to be significant (r^2^ = 0.10; F = 9.86; p<0.001) but the addition of the *m*-index did not significantly add to prediction (t = −0.46; p = 0.64).

This was the case in all fields except tobacco where there was no correlation between the number of IRVs and any of the bibliometric indicators, but the addition of the *m*-index to the model did significantly add to the model's overall predictive power (t = −2.37; p = 0.03).

## Discussion

We found a modest positive correlation between peer rankings and research citation-based metrics in all fields except for tobacco and injury. This is in accordance with a number of studies that have also found positive correlations between the different citation metrics and peer review outcomes [21], [22], [23], [24].

Our paper provides an original contribution to the debate about the relationship between peer research assessment and citation based metrics and hence to the issue of whether metrics can complement or substitute for peer review, given the acknowledged problems with the latter. In grant, promotional and institutional research ranking exercises, peer rankings are typically undertaken by a small number of assigned assessors or by relatively small panels. By contrast, in our research, we sought to involve all research-active Australian researchers in six fields in nominating up to five of their most influential peers. This process effectively produced a tally of votes about research influence that engaged a large proportion of Australian researchers active in each of these fields.

In contrast to previous comparisons of metrics with the outcomes of peer review, our method of measuring peer assessment was transparent and simple. In the studies conducted by Bornmann et al (2008a; 2008b; 2008c; 2009) and Schreiber (2008), the method of assessing peer review was not clearly described, which made it difficult to assess what components were used to evaluate each researcher's performance. There is an important advantage of field-wide peer ‘voting’ on research influence over the typical process of peer assessment involving small numbers of assessors. Most obviously, the process includes a larger and more representative sample of peers than that obtained from the assessment of a small number (sometimes only one or two) of peers. By allowing active researchers across a whole field to nominate influential researchers we obtained a ranked distribution of peer esteem in each of the six fields.

The validity of using the *h*-index and the various other evaluation metrics to describe different aspects of a researchers' research output was also tested in this paper. Our data suggests that any of the four indices could reasonably be used to complement or replace peer review for the initial stages of research and/or researcher assessment. However, we emphasise the need for a dual process to take into account the advantages and the limitations of both evaluation methods, as suggested by Moed (2007).

The poor correlation shown in the tobacco field and the negative correlation in the injury field may well be explained by the understanding of research influence for each field. For tobacco, Australia has one of the most advanced and comprehensive tobacco control policies in the world, and has seen continual falls in tobacco use since the 1960s. Hence, Australian tobacco researchers primarily evaluate influence by their impacts on government policy rather than solely rely on publishing in peer reviewed literature. While the researchers who were peer ranked highly as influential also had impressive citation metrics, there were several researchers working in more traditional areas of tobacco related research (e.g. epidemiology and biostatistics) who had very high citation metrics who were not highly ranked as influential by their peers. The negative correlation observed in the injury field arose because several researchers with very high *h*-indices received low ratings of influence and the most influential researcher in the field had a modest *h*-index. Limiting our sample to researchers with one or more influential votes restricted our range of scores and will have reduced the correlation across all fields.

In fields with strong traditions of informing government policy through the publications in the grey literature (e.g. NGO and government reports), high impact publishing will seldom be the only factor that determines peer judged research influence. Although we analysed a researcher's full list of publications (including grey literature), citations of this work are unlikely to be accurately represented by the WoS platform. Indeed, peer assessment of influence in these fields would have included these social impact aspects of research while the *h*-index only reflected citations within the peer reviewed literature. In addition, for all researchers, the citation metrics achieved seem to reflect the characteristics of the field rather than the influence of the individual researcher. Because this disparity occurred far less in the other four fields, this suggests that ratings of research influence in tobacco control and injury may be more aligned with social impact of research than in the other areas. Indeed, the modest *h*-index of the most influential researcher in the injury field reflects an output of applied research that was published in government and health service reports.

The results suggest that researchers in four of the six fields valued the work of peers who had a high publication visibility, attracted a higher number of citations and published in journals with high impact factors. This is particularly striking given that the criteria for peer nominations asked about researchers' ability to influence policy and practice, and did not explicitly mention scientific impact. Although taking into account a variety of scientific and social impact factors can only strengthen and further inform the research evaluation process, the more traditional research value of publishing still appears to play a substantial role in peer judgments about researchers' policy influence. This conclusion is also informed by the post-survey interviews conducted with the top 36 nominees in which the majority of interviewees explained they had used social *and* scientific impact criteria in their assessments of peers for nomination [16].

We also found that for most fields the addition of the *m*-index did not add to the predictive value of IRVs. This suggests that in the case of public health the use of one index is sufficient to predict a researcher's influence as rated by peers. This was in contrast to Bornmann et al (2008a) who emphasised the importance of using multiple indices to reflect both the quantity and quality (impact) of the researcher's core publications, the *h*-core. However, this finding can also be related to the nature of the *h*- and *m*-indices which has been found to be highly correlated [25].

The *h*-index was a significant predictor in 4 of the 6 fields but not in the tobacco and injury fields. The inclusion of the *m*-index did significantly add to the overall prediction in the tobacco field. From this we can speculate that the addition of further metrics that represent different aspects of research output may be needed to predict research influence via peer assessments in the tobacco field.

The most appropriate metrics for research evaluation will certainly differ between research fields. Although the fields examined in this paper share a common aim, being loosely labelled as ‘public health’, it is reasonable to assume that other research fields will require different metrics for assessing research impact. These field-specific metrics would represent those aspects of research output that are uniquely valued highly by members of that research community. As such, further research and proper consultation should take place to determine which metric, or combination of metrics, reflect each field's consideration of what form of research output are most influential. This variation should be taken into account when evaluating research performance across a disparate range of fields. However it is also likely that there are some measures of research influence that are outside the current capabilities of metrics.

The differences observed between public health fields suggest that methods of evaluating researcher impact may need to take such differences into account rather than assuming a ‘one size fits all’ model. The results presented in this paper suggest that the *h*-index, *m*-index, *m*-quotient and the new *q^2^*-index are all potentially useful metrics to evaluate research performance. Their role in assessing research performance in public health research remains to be more comprehensively assessed by similar studies in other countries and research fields.
